# Honey Enriched with Additives Alleviates Behavioral, Oxidative Stress, and Brain Alterations Induced by Heavy Metals and Imidacloprid in Zebrafish

**DOI:** 10.3390/ijms252111730

**Published:** 2024-10-31

**Authors:** Emanuela Paduraru, Roxana Jijie, Ira-Adeline Simionov, Cristina-Maria Gavrilescu, Tudor Ilie, Diana Iacob, Andreea Lupitu, Cristian Moisa, Claudia Muresan, Lucian Copolovici, Dana M. Copolovici, Gabriela Mihalache, Florin Daniel Lipsa, Gheorghe Solcan, Gabriela-Alexandra Danelet, Mircea Nicoara, Alin Ciobica, Carmen Solcan

**Affiliations:** 1 Doctoral School of Geosciences, Faculty of Geography and Geology, Alexandru Ioan Cuza University of Iasi, No. 20 A Carol I Avenue, 700505 Iasi, Romania; emanuelapaduraru19@yahoo.com (E.P.); dianaelenaiacob84@gmail.com (D.I.); mirmag@uaic.ro (M.N.); 2 Research Center on Advanced Materials and Technologies (RAMTECH), Department of Exact and Natural Sciences, Institute of Interdisciplinary Research, Alexandru Ioan Cuza University of Iasi, No. 11 Carol I Avenue, 700506 Iasi, Romania; roxana.jijie@uaic.ro; 3 Department of Food Science, Food Engineering, Biotechnologies and Aquaculture, Dunarea de Jos University of Galati, No. 47 Domnească Street, 800008 Galati, Romania; ira.simionov@gmail.com; 4 REXDAN Research Infrastructure, Dunarea de Jos University of Galati, No. 98 George Coșbuc Street, 800385 Galati, Romania; 5 Department of Biomedical Sciences, Grigore T. Popa University of Medicine and Pharmacy, No. 16 University Street, 700115 Iasi, Romania; cristina.gavrilescu@umfiasi.ro; 6 Synergy Plant Products, No. 12 Milano Street, Prejmer, 507165 Brasov, Romania; tudoriliebv@gmail.com; 7 Faculty of Food Engineering, Tourism and Environmental Protection, Institute for Research, Development and Innovation in Technical and Natural Sciences, Aurel Vlaicu University, No. 2 Elena Dragoi Street, 310330 Arad, Romania; pag.andreea@yahoo.com (A.L.); moisa.cristian@yahoo.com (C.M.); claudia.muresan@uav.ro (C.M.); lucian.copolovici@uav.ro (L.C.); dana.copolovici@uav.ro (D.M.C.); 8 Integrated Center of Environmental Science Studies in the North-Eastern Development Region (CERNESIM), Department of Exact and Natural Sciences, Institute of Interdisciplinary Research, Alexandru Ioan Cuza University of Iasi, No. 11 Carol I Avenue, 700506 Iasi, Romania; gabriela.mihalache@uaic.ro; 9 Department of Food Technologies, Ion Ionescu de la Brad University of Life Sciences, No. 3 Mihail Sadoveanu Alley, 700490 Iasi, Romania; florin.lipsa@iuls.ro; 10 Faculty of Veterinary Medicine, Ion Ionescu de la Brad University of Life Sciences, No. 8 Mihail Sadoveanu Alley, 700489 Iasi, Romania; gsolcan@uaiasi.ro (G.S.); danelet.alexandra95@yahoo.com (G.-A.D.); csolcan@uaiasi.ro (C.S.); 11 Department of Biology, Faculty of Biology, Alexandru Ioan Cuza University of Iasi, No. 20A Carol I Avenue, 700505 Iasi, Romania; 12 Center of Biomedical Research, Romanian Academy, No. 8 Carol I Avenue, 700506 Iasi, Romania; 13 Academy of Romanian Scientists, No. 54 Independence Street, Sector 5, 050094 Bucharest, Romania; 14 “Ioan Haulica” Institute, Apollonia University, No. 11 Pacurari Street, 700511 Iasi, Romania

**Keywords:** honey formulation, environmental contaminants, protective effects, combined exposure, physicochemical properties, antioxidant properties, antimicrobial activity

## Abstract

Environmental concerns have consistently been a focal point for the scientific community. Pollution is a critical ecological issue that poses significant threats to human health and agricultural production. Contamination with heavy metals and pesticides is a considerable concern, a threat to the environment, and warrants special attention. In this study, we investigated the significant issues arising from sub-chronic exposure to imidacloprid (IMI), mercury (Hg), and cadmium (Cd), either alone or in combination, using zebrafish (*Danio rerio*) as an animal model. Additionally, we assessed the potential protective effects of polyfloral honey enriched with natural ingredients, also called honey formulation (HF), against the combined sub-chronic toxic effects of the three contaminants. The effects of IMI (0.5 mg·L^−1^), Hg (15 μg·L^−1^), and Cd (5 μg·L^−1^), both individually and in combination with HF (500 mg·L^−1^), on zebrafish were evaluated by quantifying acetylcholinesterase (AChE) activity, lipid peroxidation (MDA), various antioxidant enzyme activities like superoxide dismutase and glutathione peroxidase (SOD and GPx), 2D locomotor activity, social behavior, histological and immunohistochemical factors, and changes in body element concentrations. Our findings revealed that all concentrations of pollutants may disrupt social behavior, diminish swimming performances (measured by total distance traveled, inactivity, and swimming speed), and elevate oxidative stress (OS) biomarkers of SOD, GPx, and MDA in zebrafish over the 21-day administration period. Fish exposed to IMI and Hg + Cd + IMI displayed severe lesions and increased GFAP (Glial fibrillary acidic protein) and S100B (S100 calcium-binding protein B) protein expression in the optic tectum and cerebellum, conclusively indicating astrocyte activation and neurotoxic effects. Furthermore, PCNA (Proliferating cell nuclear antigen) staining revealed reduced cell proliferation in the IMI-exposed group, contrasting with intensified proliferation in the Hg + Cd group. The nervous system exhibited significant damage across all studied concentrations, confirming the observed behavioral changes. Moreover, HF supplementation significantly mitigated the toxicity induced by contaminants and reduced OS. Therefore, the exposure to chemical mixtures offers a more complete picture of adverse impacts on aquatic ecosystems and the supplementation with bioactive compounds can help to reduce the toxicity induced by exposure to environmental pollutants.

## 1. Introduction

The functional food product market is growing due to increased consumer awareness of healthy food, the connection between nutrition and diseases, and the demand for innovative products. Efforts to enhance honey’s nutritional value with functional ingredients are ongoing, as honey can be enriched with additional components to boost its health benefits and overall dietary composition [1,2,3,4]. Introducing additives to honey not only enhances the sensory properties of honey, such as color, taste, or smell, but also significantly amplifies its pharmacokinetic, physicochemical, and microbiological effects, as evidenced by previous research [2,5,6,7,8,9]. For example, flavored honey has been proven to possess antioxidant, antiviral, antibacterial, antifungal, and hepatoprotective properties [3,5,7,8]. Sowa et al. (2019) found that polyfloral honey enriched with *Melilotus* flowers contains coumarin and that coumarin may hold therapeutic potential [8]. Additionally, honey supplemented with sea buckthorn leaf and black raspberry fruits effectively inhibited *S. aureus* biofilm formation [5]. Furthermore, a synergistic hepatoprotective effect was observed for a mixture of chestnut honey, artichoke (*Cynara cardunculus* var. *scolymus* L.), milk thistle (*Silybum marianum* L.), and borututu (*Cochlospermum angolensis* Welw.) [7]. Moreover, the usefulness of natural supplements has attracted ample interest due to their potential protective role against the adverse effects of pollutants. It has been demonstrated that sesame oil alleviates cypermethrin-induced brain toxicity [10], *Ginkgo biloba* extract provides protective benefits against mercury chloride (HgCl_2_)-induced oxidative damage [11], dietary supplementation with *Arthrospira platensis* (Gomont, 1892) may restore normal hepatic function disrupted by cadmium chloride (CdCl_2_) exposure [12], and *Turnera diffusa* (Willd) may play a protective role against testicular toxicity induced by fenitrothion (FNT) and/or hexavalent chromium [13].

The co-occurrence of metals and pesticides in aquatic environments has been demonstrated by environmental surveys [14,15,16], and their presence in food and drinking water has also been indicated by several studies [17,18]. However, little knowledge exists about their combined toxic effects and potential interactions. Studies have associated acute and chronic exposure to heavy metals and pesticides with testicular toxicity [13], hepatotoxicity [19,20], and neurotoxicity [21,22]. For instance, co-exposure to chlorpyrifos (CPF) and Cd has been found to cause synergistic injury, reducing the viability of Hep G2cells [20]. It has been observed that the combination of CPF and Cd forms a complex that promotes the bioaccumulation of metal, leading to increased toxicity. Similar synergistic impairment has been reported in zebrafish (*Danio rerio*, Hamilton, 1822) embryos due to the combination of nickel sulfate (NiSO_4_) and buprofezin [23]. Lajmanovich et al. (2019) demonstrated the synergistic toxicity of a glyphosate-based herbicide and arsenite (As (III)) mixture, which negatively affected tadpole development by increasing OS and thyroid hormone levels [24]. In a previous study, it was shown that acute exposure to deltamethrin (DM) and lead (Pb) induces damage to zebrafish, resulting in significant alterations in their behavior [22]. Conversely, the DM-associated behavioral changes and OS were mitigated in the presence of nickel (Ni) and Cd [21]. Thus, evaluating the effects of single contaminants may not fully reflect the real exposure risk to aquatic ecosystems and human health.

The limited availability of data on the toxicity of joint compound exposure concerning animal behavior and cognitive responses is an intriguing aspect worth considering [25]. Most toxicology studies predominantly focus on alterations in physiological and biochemical parameters [26], rather than on the relationship between biological and behavioral processes [22]. As a result of its complex behavioral responses, the zebrafish (*Danio rerio*) has rapidly emerged as a popular model organism for screening the effects of various toxic and bioactive compounds and the associated mechanisms [27,28]. In addition to sharing many molecular, biochemical, cellular, and physiological similarities with mammals, zebrafish have several advantages including a high reproductive rate, external fertilization, optical transparency, small size, rapid development, a short life cycle, easy husbandry, and cost-effectiveness [29,30]. Therefore, by evaluating the impact of various substances on zebrafish, valuable information for both human and fish safety can be obtained.

In our study, a stress condition was induced by exposing zebrafish to a ternary mixture of Cd, Hg, and IMI. Previous research indicates that exposure to these contaminants can result in organ and system damage in zebrafish. For instance, Cd exposure can elicit neurotoxicity, microbiota dysbiosis, disruption of thyroid endocrine and reproductive systems, and developmental abnormalities [27,31,32,33,34,35]. Xia et al. (2020) demonstrated that exposure to 5 μg·L^−1^ Cd for 7 days induced changes in locomotor activities and in microbiota diversity and richness in zebrafish [31]. Combined exposure to 10 μg·L^−1^ Cd and polystyrene beads (5 μm in diameter) for 3 weeks induced oxidative damage and inflammation in zebrafish tissues [27]. In addition, co-exposure of zebrafish adults to 0.1 μg·L^−1^ Cd and tributyltin (TBT) for 90 days can cause neurotoxicity and disruption of the thyroid system as well as developmental impairments in the offspring larvae [34]. Stronger effects were found for zebrafish exposed to ionic Cd compared to Cd-containing nanoparticles (5 nm in diameter), for the same nominal concentration of Cd (10 μg·L^−1^) [35]. Likewise, Hg exposure may lead to cumulative impacts on motor and cognitive functions, characterized by increased anxiety-related responses [36] and decreased aggressive behavior [37]. Moreover, the short-term exposure to 7.7 and 38.5 μg·L^−1^ HgCl_2_ resulted in morpho-functional alterations in zebrafish gills and changes in the expression patterns of Na^+^/K^+^-ATPase and metallothioneins [38]. The reproductive function of adult zebrafish can be impaired by exposure to environmentally relevant concentrations of Hg (0.6–15 μg·L^−1^) during early life [39]. Sun et al. (2018) revealed that exposure of zebrafish embryos/larvae to Hg in the range of environmentally relevant concentrations (1–16 μg·L^−1^) for 7 days augmented the whole-body thyroid hormone levels and altered the transcription of related hypothalamic–pituitary–thyroid axis genes [40]. Similarly, IMI treatment has been associated with adverse effects on social behavior [41] and oxidative damage [42]. For instance, low concentrations of IMI (100 and 1000 μg·L^−1^) caused intestinal histological injury, OS, an inflammatory response, and gut microbiota dysbiosis [42]. Co-exposure to 100 μg·L^−1^ IMI and 20 μg·L^−1^ polystyrene (PS) microplastics for 21 days led to growth inhibition and alterations in hepatic parameters and OS-related biochemical parameters [43]. Similarly, Hou et al. (2024) showed that exposure to 10–500 μg·L^−1^ IMI inhibited the growth of zebrafish, but also altered liver glucose metabolism and decreased plasma insulin levels [44].

Therefore, the present study aims to assess the impact of two heavy metals and one pesticide on zebrafish and to investigate the potential protective effects of HF against toxicity induced by these contaminants. Our study involved a comprehensive analysis of adult zebrafish, including their behavior, biochemical profiles, and body element concentrations, as well as histological and immunohistochemical changes. Furthermore, there is currently a lack of research on the effectiveness of HF in mitigating the toxic effects of a combination of heavy metals and pesticides.

## 2. Results and Discussion

### 2.1. Physicochemical Analysis and Antimicrobial Activity of HF

According to the literature, honey’s physicochemical and biological properties are related to the botanical and geographical origins and bee species, as well as harvesting, processing, storage, and transport conditions [45]. The physicochemical parameter values for raw polyfloral honey (control) enriched with 12 ingredients (HF) are given in Table 1. Polyfloral honey showed a moisture content of 19.46%, which is below the maximum limit (20%) set by Codex Alimentarius and European standards [46,47]. While higher moisture content was noticed in the HF (22.66%), the addition of aqueous extracts increased the water content. The amount of water present in honey may affect its quality and stability during storage; e.g., honey with higher moisture content is more prone to fermentation and the formation of acetic acid [48]. In general, honey with low moisture content has high total soluble solids [49], which is in agreement with our result. Moreover, the TSS for raw honey was similar to that reported by Albu et al. (2021), who found a TSS of 80.6 ± 1.41 °Brix [50]. The pH mean values for honey samples were acidic and relatively close, ranging between 3.93 ± 0.01 for raw honey and 4.05 ± 0.05 for HF. According to published results, a pH range of 3.2 to 4.5 is considered acceptable for honey samples and is a good indicator of honey’s stability and quality [48]. Also, both honey samples met the maximum level of acidity requirement, which is 50 meq·kg^−1^ [46]. Similar results for polyfloral honey acidity were published by Sakač et al. (2019) [48], who found an acidity level of 19.3 ± 1.88 meq·kg^−1^. As the literature describes, free acidity increases during the storage time and fermentation because the sugars and the alcohols from the honey composition are transformed into organic acid by the action of yeasts [51,52]. Another important parameter in honey quality control is electrical conductivity, which correlates with the content of ions, organic acids, and proteins [53,54]. As depicted in Table 1, the obtained mean values of electrical conductivity were below the maximum limit of 0.8 mS·cm^−1^ set by Codex Alimentarius [46] and close to the data previously published by Kunat-Budzyńska and co-authors [55]. On the other hand, with the introduction of the additives, the final product is characterized by a vibrant color and robust flavor (Figure S1 in Supplementary Materials).

The mineral content is one of the parameters used to assess the nutritional value of honey, but also an indicator of environmental pollution. According to literature data, darker honey tends to have slightly higher mineral amounts compared to lighter honey [56]. Our data support this finding, the total mineral content of honey enriched with additives accounts for 871.53 μg·g^−1^, which is 2.4 times higher than the value of polyfloral honey (361.17 μg·g^−1^). K was the most abundant macroelement in both tested samples, with a mean concentration of 238.56 μg·g^−1^ for the control sample and 534.63 μg·g^−1^ for HF, respectively. The results obtained are strongly supported by other authors’ findings [56,57,58]. Further, the Pb and Cd concentrations did not exceed the acceptable levels proposed by the World Health Organization (WHO) and the Food and Agriculture Organization (FAO) of 25 μg·kg^−1^ for Pb and 7 μg·kg^−1^ for Cd [56].

Moreover, the addition of additives enhanced the total content of phenolic compounds (2.6-fold), flavonoids (4.5-fold), and carotenoids (5.7-fold increase) compared to the control sample. Further, the flavonoid fraction of total phenolic compounds increased from 41.6% to 72.7% with the introduction of 12 additives to polyfloral honey. A similar effect of enriching honey with polyphenolic compounds was previously observed for honey supplemented with powdered sea buckthorn leaves and fruits, respectively [5]. Higher enrichment (over 400%) was reported for honey mixed with 4% fruit or 1% leaves of *Rubus* sp. [3].

The superiority of the antioxidant activity (DPPH and ABTS) of HF over raw honey was also observed, which is in agreement with other studies [2,59]. For example, a several-fold increase in the antioxidant capacity was found for creamed honey enriched with lavender (~3 times), lemon balm (~6.8 times), nettle (~3.2 times), peppermint (~5.9 times), and ginger (1.8 times) in contrast to polyfloral honey [2]. The results of Wilczyńska et al. (2017) [59] showed that the addition of cinnamon, ginger, and cardamom to polyfloral honey may cause changes in its antioxidant capacity. The highest antioxidant activity was obtained for honey flavored with cinnamon [59].

Among the 12 compounds explored, syringic acid, quercetin, ferulic acid, riboflavin, and pyrogallol were present in various amounts in both studied samples. As illustrated in Table 2, syringic acid was the dominant phenolic acid in the raw honey sample, and pyrogallol in the HF sample, while quercetin was found among flavonoids. Other authors indicate the presence of caffeoylquinic acids in rape honey enriched with mulberry leaves or fruits [1] and coumarin and *o*-coumaric acid in polyfloral honey fortified with *Melilotus officinalis* and *M. albus* [8]. Also, an increase in rutin and quercetin concentrations was observed in honey supplemented with *Sophora* flower [60], while Tomczyk et al. (2019) [1] highlight the potential of using mulberry leaves as a source of phenolic compounds for enriching honey instead of fruits.

Furthermore, the results reveal that the HF at the tested concentrations (0.5, 1, and 1.5 g·mL^−1^) does not exhibit antibacterial activity against Gram-negative and Gram-positive bacteria (Figure S2 in Supplementary Materials). Similarly, no antibacterial activity was observed against *E. coli* [5] and *P. aeruginosa* [9] for other types of flavored honey irrespective of the added additives (e.g., fruit, herbs, and spices). The lack of antibacterial activity of HF might be attributed to the relatively low contents and diversity of polyphenols and flavonoids (Table 2). Alzahrani et al. (2012) have shown that the honey antimicrobial effects decrease as the polyphenols content decreases [61]. In their study, the highest antimicrobial activity was reported for manuka honey with a total polyphenol content of 899.09 mg gallic acid per kg, followed by acacia honey (627.56 mg gallic acid per kg), wild carrot honey (503.09 mg gallic acid per kg), and lavender honey (111.42 mg gallic acid per kg) [61]. Equally important is the composition and content of polyphenols found in honey. For instance, the high antimicrobial effects of manuka honey were attributed to the presence of caffeic acid, pinocembrin, chrysin, and galangin among the polyphenols [62]. However, according to Cheung et al. (2019), the composition of phenolic compounds depends on the geographical origin, while the concentrations of the compounds are influenced by the floral source [63]. Apart from the phytochemical composition, other factors influencing the antibacterial activity of honey include its chemical composition (water content, acidity, level of H_2_O_2_, non-peroxide components or sugar concentration), origin (geographical, seasonal, and botanical), and harvesting, processing, and preservation conditions. In addition, the low concentrations used in the experiment could have played an important role in the absence of antimicrobial effect. In general, the concentrations reported as having antibacterial activity can range from less than 3% to more than 50% [64,65,66]. Thus, the effectiveness of different types of honey against bacteria can vary significantly, with differences between them being as much as 100-fold [64].

### 2.2. Effects of HF on Mixture of Heavy Metals and IMI-Induced Toxicity in Zebrafish

#### 2.2.1. Behavioral Analysis

Since animal behavior integrates effects across multiple biological levels, subtle changes can be observed following short-term exposures to low concentrations of compounds. Thus, behavioral assays have drawn increasing attention in toxicity studies, particularly in the ecotoxicology and pharmacology fields, as they offer numerous advantages in assessing the impact of various compounds on organisms [21,22]. Common behavioral assays include the evaluation of locomotor activity, conspecific interaction, aggressiveness, and various sensory responses [67]. In this context, we used both the locomotor activity and conspecific interaction assays to investigate whether sub-chronic exposure to heavy metals alone and in combination with IMI has any detrimental effects on fish locomotion parameters and on the time spent near the social stimulus. In addition, the efficiency of HF supplementation in alleviating behavioral impairments induced by the three toxicants was assessed.

**Figure 1:**
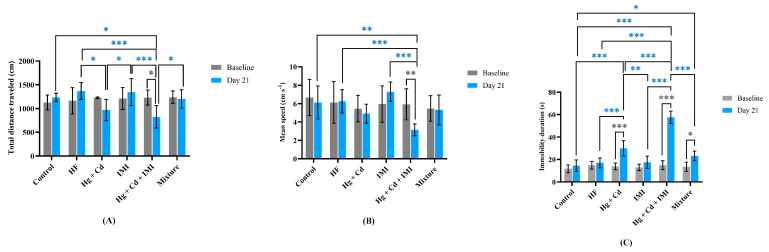
Locomotor activity of control and treated groups. (**A**) Total distance traveled (cm), (**B**) mean speed (cm·s^−1^), and (**C**) immobility duration (s). Data are expressed as mean ± SD and analyzed by two-way ANOVA followed by Tukey’s multiple comparison test. Statistically significant differences are denoted by * *p* < 0.05, ** *p* < 0.01, and *** *p* < 0.001. The experiments were repeated 2 times with 7 animals per group.

According to the results shown in Figure 1A–C, there is no significant difference in behavioral endpoints between HF-treated zebrafish and the control group, as well as in the baseline performance of animals. Instead, the sub-chronic exposure to heavy metals and IMI mixture caused significant changes in zebrafish swimming behavior compared to the control and individual contaminant groups, which were characterized by an increased period of inactivity and reduced average swim velocity and total distance traveled. For instance, the simultaneous exposure to Hg, Cd, and IMI decreased the total distance traveled by 33% and 38.6% in relation to the control (*p* = 0.01) and IMI (*p* < 0.001) groups, while decreasing the mean velocity by 48.6% and 56.9% compared with the control (*p* = 0.002) and IMI (*p* < 0.001) groups, respectively. Similarly, the results of other studies indicated that co-exposure to Pb and TiO_2_ nanoparticles [68] or carbendazim and chlorpyrifos [69] can lead to slower exploration behavior in zebrafish.

In addition, we found a significant increase in the inactivity time of the zebrafish from an average of approximately 8% of the time for both the control and HF-exposed group to an average of 24% and 12.5% for the ternary mixture (*p* < 0.001) and Hg + Cd (*p* < 0.001). Similar to our observation, zebrafish exposed to 50 µg·L^−1^ PbCl_2_ [67], 45 µg·L^−1^ IMI [70], and 0.75 mM acrylamide [71] showed behavioral changes, characterized by a significant increase in immobility duration. A previous study also reported an augmentation in immobile time and a decrease in the total distance traveled by zebrafish after 96 h exposure to various proportions of a hospital effluent [72]. On the other hand, the results indicate that the locomotor impairments induced by the ternary mixture can be partially attenuated by HF supplementation. Interestingly, there were no significant differences when comparing the velocity and distance moved by the animals from the mixture group with those from the HF and control groups, respectively. The period of inactivity for zebrafish exposed to the mixture (Hg + Cd + IMI + HF) was partially restored to the control value (*p* = 0.043). Consequently, the fish exposed to Hg, Cd, and IMI in combination with HF moved faster (1.7 times) and a longer distance (1.5 times) and had a lower immobility duration (2.5 times) than those from the Hg + Cd + IMI group. Our results are in agreement with an earlier study by Abdulmajeed et al. (2016) [72] who showed that honey supplementation may mitigate lead-induced memory and locomotor activity impairments.

**Figure 2:**
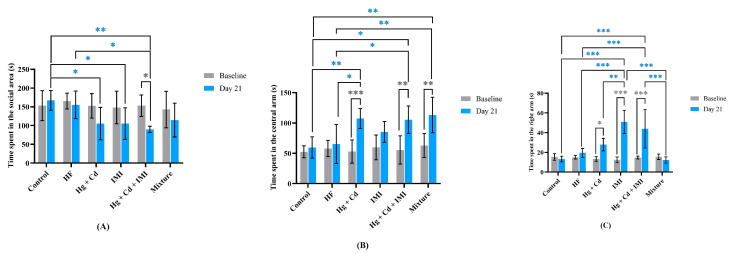
Social interaction behavior observed among zebrafish after chemical compound exposure. Time spent in the left (social area) (**A**), central (**B**), and right (**C**) arms. Data are expressed as mean ± SD (*n* = 7) and analyzed by two-way ANOVA followed by Tukey’s multiple comparison test. Statistically significant differences are denoted by * *p* < 0.05, ** *p* < 0.01, and *** *p* < 0.001. The experiments were repeated 2 times with 7 animals per group.

Furthermore, we found that exposure to singular and combined chemical treatments for 21 days impaired the zebrafish’s social behavior, as indicated by the reduced time spent in the zone near conspecific individuals compared to the control group (Figure 2A). The time spent near the social stimulus decreased by 37% when zebrafish were treated with Hg + Cd and IMI (*p* = 0.031) compared to the control group, while the highest magnitude of reduction was observed for the group exposed to heavy metals in combination with IMI (*p* = 0.004). As shown in Figure 2A,B, the HF co-treatment with chemicals restored social preference to the normal level (*p* = 0.094), but with an increase in the time spent in the central segment compared to the control group (*p* = 0.002). Similarly, the results of Robea et al. (2020) showed that the simultaneous administration of vitamin C [73] and vitamin B_12_ [74] with fipronil and pyriproxyfen can partially reverse the social interaction deficits induced by insecticides, but with an increase in the time spent in the right arm compared to the control group on day 14 of exposure.

Moreover, the zebrafish exposed to heavy metals alone and in combination with IMI showed a preference for the central segment, spending 107.4 ± 16.3 s and 105.3 ± 22.8 s, respectively. Also, the IMI (*p* < 0.001) and Hg + Cd + IMI (*p* < 0.001) groups showed a marked difference with the control regarding the time spent in the right arm (Figure 2C). Similar to our observation, valproic acid [75,76], tetrabromobisphenol A [77], silver nitrate [78], bisphenol S and estradiol [79], and antidepressant [80] treatments induced social interaction deficits in zebrafish.

#### 2.2.2. Biochemical Analysis

Biochemical responses of organisms are frequently used as early warning signs of environmental contamination [43]. The imbalance between the production and elimination of reactive oxygen species (ROS) in tissues leads to the appearance of OS, which is a key factor for many chronic diseases like neurological, metabolic, and cardiovascular disorders [70,81]. However, organisms have evolved a complex system of defense to neutralize ROS, consisting of a network of enzymatic (e.g., SOD, GPx, CAT, etc.) and non-enzymatic (e.g., vitamin C, uric acid, vitamin E, melatonin, etc.) antioxidants. The antioxidants can act at various levels, inhibiting the formation of free radicals, scavenging them, and repairing or removing the oxidized biomolecules [81]. Antioxidants are substances that counteract the deterioration caused by oxidants such as O_2_, OH^−^, superoxide, and/or lipid peroxyl radicals [82]. Cells have a defense system against oxidative damage, which includes free radicals and other protective agents such as catalase, SOD, peroxidase, ascorbic acid, tocopherol, and polyphenols [83].

Honey from different floral origins in various countries has been shown to possess high antioxidant properties [82]. The antioxidant activity of honey is primarily attributed to phenolic acids [84] and flavonoids [85,86,87]. Antioxidant agents stimulate biomolecules like carbohydrates, proteins, lipids, and nucleic acids [83]. This stimulation ultimately triggers an antioxidant response in cells. Honey and honey infused with herbs have been found to exhibit strong antioxidant activity, and this capacity contributes to the prevention of several acute and chronic disorders [83,88]. The precise antioxidant mechanism remains unclear, but proposed pathways include scavenging of free radicals, donation of hydrogen atoms, chelation of metal ions, and the role of flavonoids as substrates for neutralizing hydroxyl and superoxide radicals [83].

**Figure 3:**
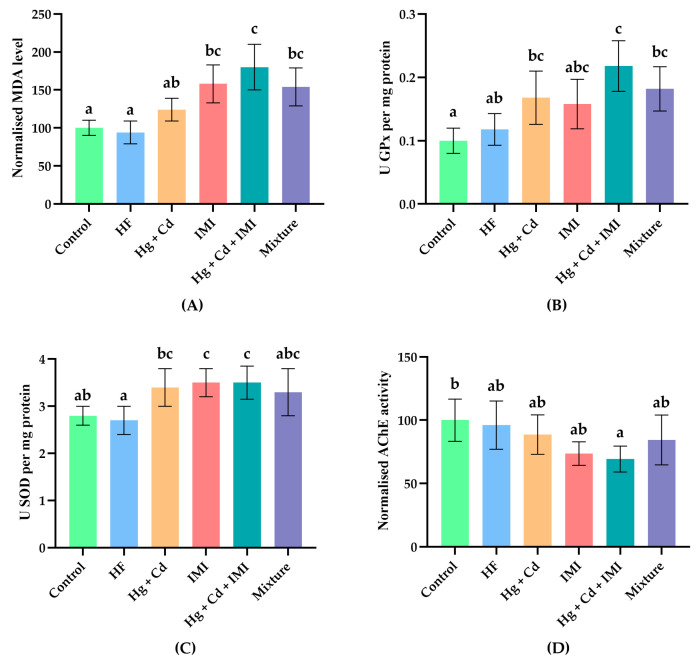
MDA level (**A**), GPx (**B**), SOD (**C**), and AChE (**D**) activities in zebrafish following treatment with Hg (15 µg·L^−1^), Cd (5 µg·L^−1^), and IMI (0.5 mg·L^−1^) alone and in combination with HF (500 mg·L^−1^) for 21 days. Data are expressed as mean ± SD (*n* = 5 animals per group with two replicates), and bars sharing the same letter indicate no significant differences at the level of *p* < 0.05 using one-way ANOVA followed by Tukey’s multiple comparison test.

The present study aims to evaluate the single and combined sub-chronic effects of IMI, Hg, and Cd treatments on selected OS parameters and AChE activity and to determine whether HF co-administration can alleviate contaminant-induced OS. To assess the oxidative damage induced by pollutants in zebrafish, we measured the activities of SOD and GPx, as well as the MDA contents. As is described in the literature, SOD and GPx are the body’s first line of defense against the toxic effects of ROS, while MDA is a marker of oxidative damage caused by these chemical compounds against zebrafish [74]. In addition, the AChE is a key nervous system enzyme often employed as a biomarker for evaluating the effects of various environmental pollutants on aquatic organisms [89]. Figure 3A–D show no significant difference in SO parameters between HF-treated (500 mg·L^−1^) zebrafish and the control group; in contrast, exposure to IMI treatment for 21 days increased MDA content (*p* = 0.003) and SOD activity (*p* = 0.047).

In a previous study, neotropical fish (*Prochilodus lineatus* Valenciennes, 1837) exposed to different IMI concentrations showed liver, gill, kidney, and brain lipid peroxidation as well as increased SOD activity in the liver and gills [90]. Also, Ge et al. (2015) showed that 5 mg·L^−1^ IMI treatment markedly increased MDA content and SOD activity in zebrafish liver at day 14 [91]. Moreover, sub-chronic exposure to heavy metals caused a significant increase in MDA level (*p* = 0.003) and GPx activity (*p* = 0.047), which were about 1.2- and 1.7-fold higher than those in control fish, respectively. Similar to our results, various reports demonstrated that HgCl_2_ [92], CH_3_HgCl [93], CdCr_2_ [94], and simultaneous CdCl_2,_ and HgCl_2_ [95] exposure induced lipid peroxidation.

The ternary mixture affected not only the main parameters related to the OS but also the AChE activity. As shown in Figure 3A–D, sub-chronic exposure to heavy metals and IMI promoted a significant inhibition of AChE activity (−30.7% compared to the control group) in the fish brains and a remarkable elevation in antioxidant enzyme activities (*p* = 0.047 for SOD and *p* < 0.001 for GPx) and MDA level (*p* < 0.001) in fish tissues, respectively. According to our findings, an earlier study by Rosales-Pérez et al. (2022) showed that acute exposure to a mixture of contaminants from a hospital effluent markedly inhibited the AChE activity and increased antioxidant enzyme activities (e.g., SOD, CAT, and GPx) in fish brains [96].

The changes in AChE activity concerning behavioral observations provide valuable insights. However, a more comprehensive investigation is needed to understand the underlying mechanisms. The partial reduction in AChE inhibition with HF supplementation suggests reversibility, raising the possibility of adaptive mechanisms mitigating neurotoxic effects in zebrafish. The preservation of swimming behavior despite inhibited AChE activity may point to the activation of compensatory motor control strategies.

In addition, Abdulmajeed et al. (2016) and Azman et al. (2015) found that honey supplementation reduced anxiety, memory impairment, decreased locomotor activity, and OS levels in rats [72,97]. In our study, co-administration of HF partially normalized OS markers in zebrafish, thus indicating a protective role against oxidative damage. Furthermore, the cholinergic system has a critical role in mediating locomotor responses to novel stimuli and facilitating spatial memory, highlighting the impact of disruptions to AChE breakdown due to compromised AChE activity [32,94,95]. While our research found that metal concentrations and pesticides negatively impacted AChE activity, it is essential to note that some studies, such as that of Bui Thi et al. (2020), have reported increased AChE activity in fish following heavy metal exposure [67]. This variability underscores the need for further exploration of the context-dependent compensatory mechanisms that zebrafish may employ in response to environmental neurotoxins, which can enhance our understanding of their resilience to neurotoxic stress at both molecular and behavioral levels.

In addition, the co-administration of HF attenuated ternary mixture-induced changes in OS parameters and AChE activity. For example, in rats, honey supplementation ameliorated the enhanced MDA level and normalized the SOD activity [98]; meanwhile, in the case of AChE, Tualang honey improved cholinergic transmission, protecting against hypoxia-induced neuronal damages [99].

#### 2.2.3. Zebrafish Body Element Concentrations

Cu, Fe, Zn, Mn, Ni, Mg, Na, and Ca constitute essential elements crucial for the optimal functionality of various cellular enzymes and proteins [100,101]. The impact of bioelements on biological processes can turn toxic when their concentrations exceed specific thresholds [100,102]. Notably, certain heavy metals possess the potential to influence metal ion dynamics within aquatic environments, prompting a growing interest in evaluating the efficacy of dietary products in mitigating pollutant toxicity [103,104,105,106].

**Figure 4:**
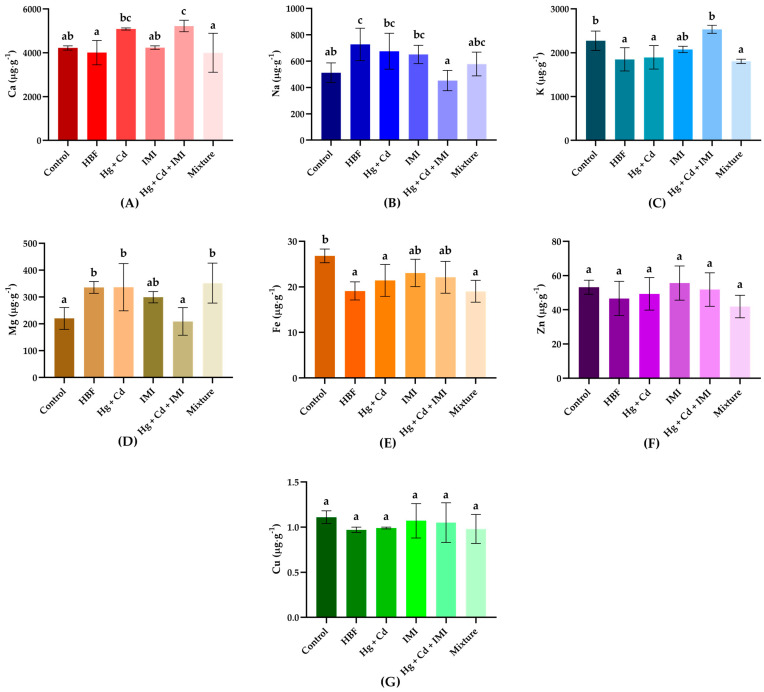
Concentration (μg·g^−1^ wet weight) of essential elements ((**A**)—Ca; (**B**)—Na; (**C**)—K; (**D**)—Mg; (**E**)—Fe; (**F**)—Zn; (**G**)—Cu) involved in biological structures and biochemical processes analyzed from entire fish body mass and reported as mean ± SD (*n* = 5 animals per group), with bars sharing the same letter indicating no significant differences at the level of *p* < 0.05 using one-way ANOVA followed by Tukey’s multiple comparison test.

In general, honey or honey-based products are described by elevated concentrations of essential elements [45,101]. In the context of our study, the administration of HF produced significant effects on some of the elemental composition of the zebrafish body (Figure 4A–G). This intervention led to a reduction in Fe^+^ levels while elevating the concentrations of Mg^+^ and Na^+^. This outcome underscores the vital physiological role of Na^+^ in nerve transmission [107] and the enhanced metabolism of Mg^+,^ a factor of particular importance [108]. The observed decrease in Fe^+^ levels suggests a potential regulatory effect on Fe^+^ metabolism.

Our study indicates a 10% decrease in Na levels within the zebrafish group exclusively exposed to neonicotinoid insecticides and heavy metals. For example, Araujo et al. (2022) showed a significant reduction in Na levels after 96 h in carbamazepine (CBZ) + acetamiprid (ACT) and ACT + Cd groups [109].

Regarding K levels, no significant differences were observed in the group exposed to the mixture compared to the control; these findings imply that the losses of K^+^ and Ca^2+^ were proportional to the ionic disparities between the fish plasma and the surrounding water, suggesting a common mechanism of acute toxicity [110]. The previously mentioned finding is in line with the finding of Araujo (2022), who showed that CBZ + ACT and CBZ + ACT + Cd treatments did not result in significant differences compared to the control group after 96 h exposure [109]. Similarly, there was no significant difference between K levels in the control groups and zebrafish exposed to 2 mg·L^−1^ of Cr [VI] for 60 days [111].

Our study further demonstrated that exposure to a combination of Hg and Cd led to a 20% increase in Ca levels, while exposure to Cd, Hg, and IMI resulted in a 24% increase in Ca levels. The present study aligns with the findings of Siblerud et al. (2019) [112], linking Hg to elevated intracellular Ca concentrations. Increased Ca levels were measured in zebrafish exposed to CBZ, ACT, Cd, and their combined treatments [109]. Similarly, Shaw et al. reported an increase in Ca amount in the zebrafish treated with 2 mg·L^−1^ of Cr [VI] [111].

The levels of Cu and Zn did not differ significantly between the control group and the fish exposed to Cd, Hg, and IMI; these substances did not cause considerable changes in Cu and Zn metabolism. Devarapogu and Asupatri (2023) suggested a potential role for Zn in alleviating Cd toxicity [113]. Contrary to our results, significant differences in Zn and Cu levels compared to controls were measured in zebrafish exposed to CBZ, ACT, Cd, and their binary combination [109], as well as for Cr [VI]-treated zebrafish [111]. However, further analysis is necessary to understand the possible interactions and effects of mixtures on essential elements comprehensively.

#### 2.2.4. Evaluation of Histologic and IHC Lesions

**Figure 5:**
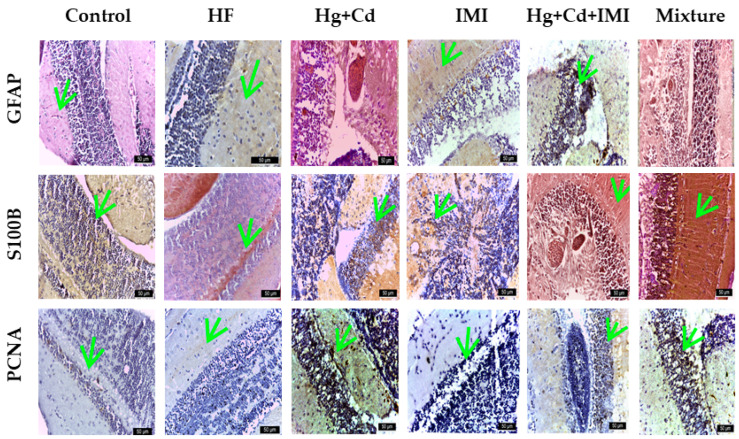
IHC expression of GFAP, S100B, and PCNA markers in the optic tectum for the control and exposed groups green arrow marks IHC positive cells (*n* = 5 animals per group; scale bar = 50 µm).

Zebrafish from the control and HF groups did not present histologic changes in the studied nervous system areas. The lesion severity score was (-). Contrarily, the optic tectum in fish exposed to IMI and Hg + Cd + IMI showed severe spongiosis in the neuronal layer, edema, and vacuolization of the neuropil with multiple areas of extravasated erythrocytes, as shown in Figure 5.

In addition, degenerative changes of neurons and neuropil and focal aggregation of microglial cells were recorded in the optic tectum for heavy-metal- and IMI-exposed animals with the lesion score (+++) (Table S2 in Supplementary Materials). The Hg + Cd group had the lesion score (+). The intensity of DAB anti-GFAP staining was significantly higher in IMI, Hg + Cd, and Hg + Cd + IMI (+++). An intermediate intensity score (++) was observed in the optic tectum of the group exposed to Hg, Cd, and IMI. Thus, GFAP expression is confirmed to increase with astrocytic emergence and activation; it is commonly adapted as a marker to study astrocyte response to various physiological and pathological conditions. Astrocytes play a significant role in nervous system development, synaptic transmission, and nerve tissue repair.

Researchers identified GFAP as a sensitive indicator of early response to neurodegenerative injury [114], and it can be detected in astrocytes even in the absence of neuronal death [115]. The severity of the injury correlates with the intensity of GFAP expression in reactive astrocytes [116,117]. In various central nervous system (CNS) disorders, activated astrocytes exhibit an increased GFAP expression due to elevated mRNA levels and increased cytoskeletal GFAP proteins. It is important to note that the elevation of GFAP levels alone is insufficient for classifying reactive astrocytes, suggesting that increased GFAP levels occur in response to pathological stimuli and regional astrocyte differences. Furthermore, initial GFAP expression in astrocytes can occur following physiological stimuli [116]. Astrocytes, as integral CNS cells, play crucial and diverse roles [118]. They are involved in metal uptake and sequestration, thereby preventing the accumulation of metals in the CNS at toxic concentrations [119,120]. Additionally, astrocytes protect neurons against various insults, including excess glutamate [121,122] and heavy metals, which are a primary source of neurotoxicity leading to neurodegenerative changes. The influx of heavy metals affects astroglial homeostatic and neuroprotective cascades, including the glutamate/GABA–glutamine complex, antioxidant systems, and energy metabolism. The astrocytes produce nerve growth factor and S100 protein, essential for neurite elongation and outgrowth [123,124]. Activated astrocytes are involved in angiogenesis, a crucial process in CNS development and repair that relies on physical interaction between astrocytes and endothelial cells. Notably, endothelial cells separated from astrocytes do not form capillaries [125,126].

In the control and HF groups, there was a noticeable difference in the reaction to anti-GFAP DAB staining. The HF group showed a more intense response. Additionally, the scoring indicated a result of (-) in the control group and a result of (+) in the HF group.

Figure 5 demonstrates the presence of the S100B protein in all experimental groups. The mesencephalic optic tectum predominantly contains S100B in nerve fibers rather than nerve cells. The protein showed immunoreactivity across all the fiber profiles, which run from the bottom to the top of the optic tectum perpendicular to its outer surface. Additionally, immunoreactivity in the medial and lateral regions of the cerebral valve was noticed. The inner surface of the optic tectum, adjacent to the tectal ventricle, contained S100B-positive ependymal and subependymal cells. The bulkier and rounder shapes of ependymal cells set them apart and make them easily identifiable.

In contrast, subependymal (glial) cells exhibited elongated radial processes that extended through the optic tectum and reached the surface of the pial membrane (*pia mater*). In the control group, the observed immunoreactivity was rated as (+), while in the HF group, it was (++). The mixed group displayed intense positivity (+++) in cytoplasmic and extracellular regions, whereas moderate positivity (++) was detected in groups exposed to individual or combined chemicals (IMI, Hg + Cd). Furthermore, the dorsal and lateral sections of the *torus longitudinalis* were lined with S100B-positive ependymal cells, and the nerve fibers forming the commissure in the ventral region of the *torus longitudinalis* were also S100B-positive. The presence of extracellular protein S100B might have a role in regulating tissue development and facilitating regeneration or repair processes.

In the assessment involving IHC staining for PCNA (Figure 5), a marker indicative of the nuclei of dividing cells, it was observed that the PCNA marker was evident in both the control group and the group exposed to HF (++). The neurotoxic effects reduced the PCNA staining in the IMI-exposed group, indicating a decrease in cell proliferation. An intense (+++) reaction was recorded in the Hg + Cd group, a moderately intense (++) reaction in the IMI and mixture groups, and a weakly positive (+) reaction in the Hg + Cd + IMI (refer to Table S2 in the Supplementary Materials). The toxicities of Cd and Hg may be mitigated, prompting the body to stimulate cell regeneration and potentially repair the damage.

**Figure 6:**
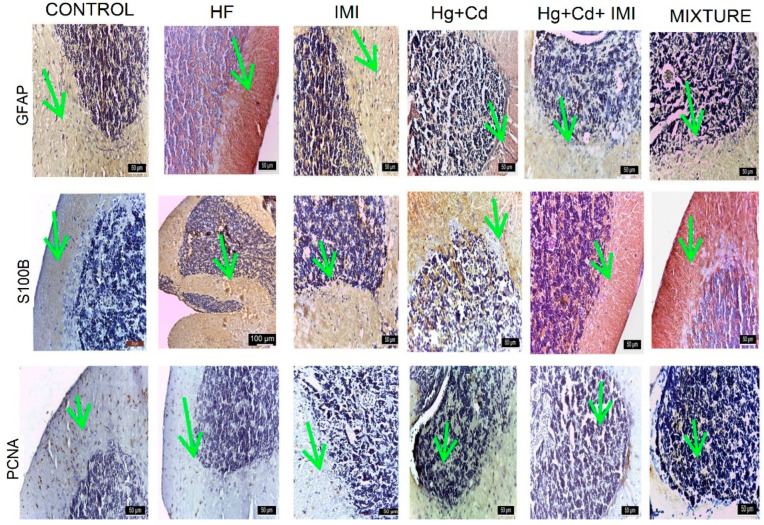
IHC expression of GFAP, S100, and PCNA markers in the cerebellum for the control and exposed groups, green arrow marks IHC positive cells (*n* = 5 animals per group; scale bar = 50 µm).

In the cerebellum, IMI and Hg + Cd + IMI cause neuronal degeneration, spongiosis, and vacuolation with focal aggregation of glial cells and damage to granule neurons. A reduction in cell density, depletion of the nuclear area, and chromatin condensation in granular cells were also identified (Figure 6).

Vacuolation of grey and white matter, necrotic cells, and the presence of tissue edema that caused large lesions due to OS were observed during the analysis. Significant damage was documented in the cerebellar cortex after exposure to Hg + Cd, resulting in a neuronal loss lesion score of ++ (Table S3 in Supplementary Materials). The cerebellum showed slight vacuolization and degeneration of cells in the Purkinje cell layer with a score of (++). No changes were recorded in the control and HF groups, while in the mixture group, the lesion score was (++).

The intensity of DAB anti-GFAP staining in the cerebellum was as follows: significantly more intense, receiving a score of (+++) for the mixture group; intermediate intensity (++) for the Hg + Cd, Hg + Cd + IMI, and IMI-exposed groups; and reduced intensity for the control group (Table S3 in the Supplementary Materials). The distribution pattern of the S100B protein in the cerebellum differs from that of other central nervous segments. It is mainly localized in neurons and less in glial cells. The cerebellar body showed S100B protein in small neurons, localized primarily in the superficial layer. In addition, neurons forming the deep cerebellar nuclei were also immunoreactive for S100B. Purkinje neurons localized in the basal zone showed a strong reaction for S100B protein in both the perikaryon and the dendritic arbor. Intense reactions were observed in the group exposed to a ternary mixture of S100B, and reactions were rated as medium (++) in HF and Hg + Cd and low in the control group. The PCNA reaction was most intense (+++) in the Hg + Cd group, followed by a medium reaction (++) in the mixture, HF, and control groups, and low in the Hg + Cd + IMI group.

**Figure 7:**
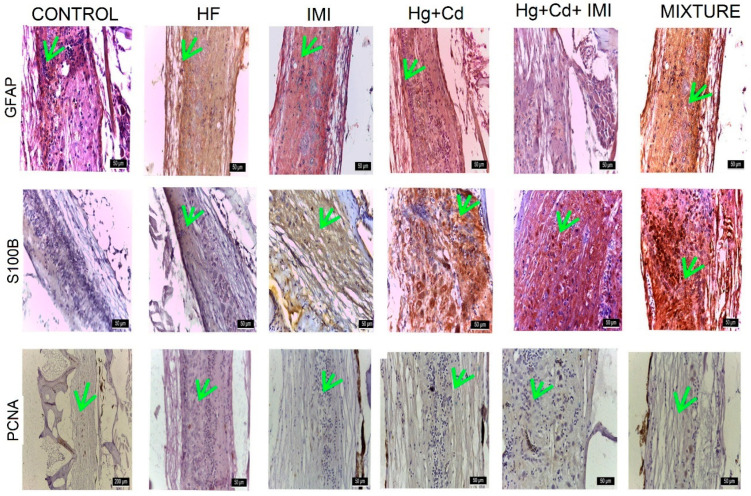
IHC expression of GFAP, S100, and PCNA markers in the spinal cord for the control and exposed groups, green arrow marks IHC positive cells (*n* = 5 animals per group; scale bar = 50 µm).

In the spinal cord, the intensity of DAB anti-GFAP staining was as follows: significantly more intense (+++) for the mixture group; of intermediate intensity (++) in Hg + Cd, Hg + Cd + IMI, and IMI-exposed groups, and reduced (+) in the control group, as shown in Table S4 in the Supplementary Materials. The distribution pattern of S100B protein in the cerebellum differs from that of other central nervous segments. It was localized mainly in neurons and less in glial cells. The reaction to PCNA was most intense (+++) in the Hg + Cd group, followed by a medium reaction (++) in the mixture, HF, and control group, and low (+) in the Hg + Cd + IMI group (Figure 7).

In the current investigation, the exposure of zebrafish to contaminants triggered a cascade of responses characterized by heightened oxidative and nervous system stress. This radical interference led to membrane damage, potential impairment, and morphological alterations in mitochondrial cristae, culminating in disrupted adenosine triphosphate (ATP) production and a subsequent decrease in intracellular ATP content [127]. The observed mitochondrial effects may have substantial implications for neurological function. Neurons have the highest energy requirements of all somatic cells. They are the largest consumers of oxygen (O_2_)—the brain consumes O_2_ at a rate ten times higher than any other tissue, making it much more vulnerable to oxidative damage [128,129]. In addition, cells have difficulty repairing the distal ends of dendrites and axons due to their distance from the cell body [129]. The heightened impact is associated with oxidative injury to mitochondria, which play a significant role in upholding the health of axons [130]. Although considerable efforts have been dedicated to the IMI toxicity on aquatic organisms, only a few data are on apoptosis and immunotoxicity [131]. IMI in common carp moderately induces apoptosis in the brain but also induces severe histopathological changes, inflammation, and OS in the gills, liver, and brain, in agreement with our results. The mechanisms of the activation of some biomarkers (iNOS, 8-OHdG, TNF-α) and expression of some apoptotic genes (caspase 3) during pesticide intoxication have been reported [132,133]. Cd also induces caspase-mediated apoptosis and necrosis in cortical neurons [127]. In addition, Cd exposure can cause a decrease in several critical brain enzymes, including AChE, acid phosphatases, alkaline phosphatase, ATPase, and catalase [134]. The observed neurotoxic effects of MeHg on zebrafish embryos could be because MeHg is known to disrupt microtubule formation [135], which could profoundly affect cell division. The toxic impact of MeHg could also be because it negatively affects mitochondrial function, thus decreasing the metabolic capabilities of cells. A study in adult zebrafish showed that exposure to 15 µg·L^−1^ of MeHg under ambient conditions caused uncoupling and mitochondrial oxidative phosphorylation in skeletal muscle [136].

The GFAP and S100B markers were selected for the protein homology studies between human, mouse, and zebrafish orthologs. GFAP (astrocyte intermediate filament), whose expression increases as astrocytes arise (astrocyte activation), is commonly adopted as a marker to study the reaction of astrocytes in various situations of physiological and pathological conditions, playing a vital role in nervous system development, synaptic transmission, and nerve tissue repair. In the present study, the expression of GFAP was considerably increased in the IMI-treated group and discrete in the control group. The observed changes may suggest heightened dopaminergic and serotonergic activity in the brain, along with initial behavioral shifts [137]. This study noted strong and extensive expression of S100B and moderate, discrete expression of GFAP in the zebrafish’s central and peripheral nervous systems, aligning with previous research findings [138]. For instance, S100B is recognized for its direct interaction with Dbf2-related nuclear protein kinase (NDR kinase), inhibiting the recruitment of NDR kinase substrates [138,139,140]. S100B deactivates advanced glycation end products (RAGEs) by binding with vital fibroblast growth factor and its receptor [139,140]. Extracellular S100 proteins play a role in regulating the apoptosis, proliferation, differentiation, and migration of various cell types, such as monocytes, macrophages, neutrophils, lymphocytes, myoblasts, epithelial cells, endothelial cells, smooth muscle cells, neurons, and fibroblasts. Additionally, besides their function as Ca-binding proteins, S100 proteins were later identified as DAMP molecules [141] associated with cell death and tissue damage, triggering rapid inflammatory responses or producing biologically active molecules [142,143]. S100B protein’s presence in the adult zebrafish nervous system contributes to intracellular Ca homeostasis regulation as a triggering or activating protein. It also exhibits neurotrophic activity, suppresses phosphorylation, induces apoptosis [144], and maintains cytoskeletal stability [145]. Furthermore, S100B protein is produced, stored, and released by astrocytes, tanycytes, oligodendrocytes, and radial cells and exerts paracrine and autocrine effects on neurons and glia. It has been shown that S100B expression defines a late developmental stage, after which the GFAP-expressing stage of development loses its potential as neuronal stem cells [146]. Since S100 protein continues to be deposited in these regions of adult zebrafish, and subventricular zone glial cells play an essential role in adult zebrafish neurogenesis, S100 protein could be involved in this function, which is maintained in zebrafish during their entire lifespan [147,148]. Using immunohistochemical staining for PCNA, a marker for the nuclei of cells undergoing cell division is expressed in developing zebrafish [149]. The expression of PCNA was significantly decreased in animals exposed to different concentrations of MeHg [150]. The neural toxicity of MeHg tested in this study caused a decrease in PCNA staining, suggesting a decline in cell proliferation. Smith et al. (2010) observed that in adult zebrafish, the telencephalic cell density was significantly decreased at all MeHg exposures during development [151]. These observations are similar to those reported by Yang et al. (2007), where MeHg exposure significantly impaired zebrafish development [152].

Higher production of antioxidants can act as a protective shield during oxidative damage [153,154,155]. OS has been implicated in the pathogenesis of neurotoxic mechanisms of several pesticides because the brain has a higher susceptibility to oxidative damage. OS occurs due to a mismatch between pro-oxidant and antioxidant systems. The resulting OS can cause oxidative damage to lipids, nucleic acids, and proteins [137]. The combination of heavy metals and IMI causes degenerative changes in the optic tectum and cerebellum and is somewhat reduced in the spinal cord. The use of GFAP, S100, and PCNA markers highlights the lesions in groups exposed to pesticides and heavy metals and their amelioration by reducing the effect of OS.

## 3. Materials and Methods

### 3.1. HF Composition and Analysis

#### 3.1.1. HF Composition

The commercial HF was received directly from the producer. This product contains a mixture of 90.39% polyfloral honey and 9.61% other natural ingredients, such as royal jelly, Spirulina *Arthrospira platensis*, extracts of *Arctium lappa* L., *Matricaria chamomilla*, *Eleutherococcus senticosus*, *Silybum marianum*, *Panax ginseng*, *Astragalus membranaceus*, *Boswellia serrata*, and essential oils from *Matricaria chamomilla*, *Salvia sclarea*, and *Salvia officinalis.* Adding natural ingredients to the polyfloral honey resulted in a notable alteration in its color, causing it to transition to a deep shade of blue (as depicted in Figure S1 in Supplementary Materials). Of the 12 constituents, the royal jelly [156], *Eleutherococcus senticosus* [157], *Arctium lappa* L. [158], *Matricaria chamomilla* [159,160], and *Silybum marianum* [161] are reported to exhibit neuroprotective effects and reduce oxidative damage. Polyfloral honey was obtained from the apiary located in the Bunesti-Viscri region (Romania) in the 2021 bee-keeping season. For transparency and to avoid any potential conflicts of interest concerning honey-based products, the brand name of the producer has been kept confidential. This product was chosen for this study to simulate a realistic scenario.

#### 3.1.2. Physicochemical Analysis

The moisture content (%), total soluble substances (°Brix), and free acidity (mequiv·kg^−1^) for polyfloral honey without (control) or with additives (HF) were assessed following the guidelines provided by the Codex Alimentarius Commission—Revised Codex Standard for Honey Codex Stan 12-1981, Rev. 1 (1987), Rev. 2 (2001) [46]. Furthermore, the pH and conductivity (mS·cm^−1^) were measured using multiparameter analysis equipment from XS Instruments, Carpi, Italy.

#### 3.1.3. Determination of Mineral Elements

The mineral analysis was conducted at the laboratory of the Faculty of Food Science and Engineering, Dunarea de Jos University of Galati, Romania. In preparation for the quantification analysis, minerals from polyfloral honey samples were extracted. This process involved treating approximately 0.5 g of honey samples (with and without additives) with a mixture of 5 mL HNO_3_ Suprapur^®^ of 65% concentration (certified Merck, Darmstadt, Germany) and 2 mL H_2_O_2_ EMSURE^®^ of 30% concentration (certified Merck, Darmstadt, Germany), followed by digestion using a microwave-assisted pressure digestion system (TOPwave, Analytik Jena, Jena, Germany) The mineralized samples were then transferred into decontaminated polyethylene flasks (50 mL volume) and diluted with ultrapure water (18.2 MΩ·cm^−2^, equipment LaboStar^™^ UV 4 Siemens Water Technologies, Barsbüttel, Germany). The treated samples were processed and then analyzed for the quantification of Calcium (Ca), potassium (K), magnesium (Mg), and sodium (Na) through the flame atomic absorption spectrometry technique (FL-AAS) using an air–acetylene–nitrous oxide flame. Furthermore, the determination of copper (Cu), iron (Fe), manganese (Mn), and zinc (Zn) was performed using the graphite furnace atomic absorption spectrometry technique (GF AAS). The ContrAA 700 equipment manufactured by Analytik Jena, Jena, Germany, was utilized to conduct these measurements. Calibration curves were established through the preparation and analysis of a reference standard solution, ICP multi-element standard solution IV with 23 elements in diluted HNO_3_ (Certipur^®^, 1000 mg·L^−1^, Merck, Darmstadt, Germany). Each sample underwent triplicate analysis, and the metal ion content was determined at the mg·g^−1^ level.

#### 3.1.4. Determination of Total Phenolic Content (TPC), Total Flavonoid Content (TFC), and Total Carotenoid Content (TCC)

The TPC was determined according to the method proposed by Csakvari et al. (2021) [162], while the TFC and TAC were measured as outlined by Rababah et al. (2014) [163], for 4% solutions of honey in distilled water. The results for TPC and TFC were expressed in gallic acid equivalents (GAE) and quercetin equivalents (QE) per 100 g sample, respectively, using appropriate calibration curves with standard solutions (gallic acid monohydrate, Sigma-Aldrich Chemie GmbH, Taufkirchen, Germany).

#### 3.1.5. Quantification of the Total Antioxidant Capacity (TAC)

The total antioxidant activity was determined by 1,1-diphenyl-2-picrylhydrazyl (DPPH; Sigma-Aldrich Chemie GmbH, Taufkirchen, Germany) and 2,2′-azino-bis-(3-ethylbenzothiazoline-6-sulfonic) acid (ABTS; Sigma-Aldrich Chemie GmbH, Taufkirchen, Germany) assays, as earlier reported by Bogdan et al. (2021) [164] and Csakvari et al. (2021) [162]. The results were expressed as mg gallic acid equivalents (GAE) per 100 g of sample and mg Trolox equivalents (TE) per 100 g of sample, respectively.

#### 3.1.6. RF-HPLC Analysis of Honey Flavonoids and Other Phenolic Derivates

The identification and quantification of phenolic compounds were performed using an ultra-high-performance liquid chromatograph (Nexera X2, Shimadzu, Tokyo, Japan) equipped with a diode array detector (M30A, Shimadzu, Tokyo, Japan) and a Nucleosil 100-3 C18 reversed-phase column (4.0 mm column inner diameter × 125 mm column length, 3 µm particle size, Macherey-Nagel GmbH, Düren, Germany). The column temperature was maintained at 30 °C, and the flow rate was 0.5 mL per min. The solvents used for the chromatographic elution consisted of ultrapure water (18.2 MΩ·cm^−2^, equipment Adrona Crystal EX, Adrona, Riga, Latvia) with 0.1% trifluoroacetic acid (A) (Sigma-Aldrich Chemie GmbH, Taufkirchen, Germany) and acetonitrile (B) (Sigma-Aldrich Chemie GmbH, Taufkirchen, Germany). The chromatographic elution program used was as follows: 5% B, then 42% B for 5 min, followed by 35% B, 5% B in 35 min. The HF was diluted 1:5 with 96% ethanol (CH_3_CH_2_OH, EMSURE^®^, Merck KGaA, Darmstadt, Germany) and then filtered using 25 mm syringe filters (Labbox Labware S.L., Barcelona, Spain). Then, the HF was injected at a volume of 10 µL and the spectra were acquired between 200 and 600 nm. The standards used were ascorbic acid, pyrogallol, gallic acid, riboflavin, rutin, caffeic acid, vanillic acid, syringic acid, *p*-coumaric acid, catechin, rosmarinic acid, ferulic acid, quercetin, and kaempferol (Sigma-Aldrich Chemie GmbH, Taufkirchen, Germany). Polyphenols were identified by comparing the retention times and UV-Vis spectra with the previously mentioned standards.

#### 3.1.7. Antibacterial Assay

The screening of HF for antibacterial activity was performed by the disk diffusion approach. It was performed using six standard strains of microorganisms from Microbiologics (Saint Cloud, MN, USA), including three strains of Gram-negative bacteria [*Escherichia coli* (ATCC 8739), *Salmonella enteritidis* (ATCC 13076), *Pseudomonas aeruginosa* (ATCC 27853)] and three strains of Gram-positive bacteria [*Staphylococcus aureus* (ATCC 25923), *Enterococcus faecalis* (ATCC 19433), *Listeria monocytogenes* (ATCC 13932)]. The strains were first grown on Mueller–Hinton Agar (MHA, Scharlab, Barcelona, Spain) for 24 h at 37 °C in order to develop single colonies, and then one colony was inoculated in 10 mL of Mueller–Hinton Broth (MHB, Carl Roth, Karlsruhe, Germany) under shaking conditions (160 rpm) at 37 °C for 24 h. After incubation, an inoculum of 0.5 MacFarland turbidity standards (~2 × 10^8^ CFU·mL^−1^) of each strain was prepared, and 200 µL was surface-spread on MHA plates. Antibiotic assay discs (Whatman™, Cytiva, Marlborough, MA, USA) of 6 mm in diameter, saturated with 20 µL of 0.5 mg·mL^−1^, 1 mg·mL^−1^, and 1.5 mg·mL^−1^ HF solutions prepared with sterile distilled water, were placed on the surface of inoculated plates. As a reference, two different antibiotics (Bio-Rad, Marnes-la-Coquette, France) for each bacterial strain were used as presented in Table S1 (Supplementary Materials). The plates were incubated at 37 °C, and the zone of inhibition against the test microorganism was measured after 24 h. The test was carried out in triplicates.

### 3.2. Zebrafish Protocols and Analysis

#### 3.2.1. Chemicals and Reagents Used for Exposure Protocols and Biochemical Analysis

A standard solution of Hg Certipur^®^ (1000 mg·L^−1^), a standard solution of Cd Certipur^®^ (1000 mg·L^−1^), nitric acid 65% Suprapur^®^ (HNO_3_, 100441), hydrogen peroxide 30% Perhydro^®^ (H_2_O_2_, 107210), phosphate-buffered saline (PBS, P4417-50TAB), acetylthiocholine iodide (ATChl, A5751), Tris hydrochloride solution (Tris-HCl) solution (Trizma^®^, T2819), 5,5′-dithiobis(2-nitrobenzoic acid) (DTNB, 322123), Bovine Serum Albumin (BSA, A8022-10G), Bradford Reagent (B6916), Lipid Peroxidation Assay Kit (MDA, MAK085), Glutathione Peroxidase Cellular Activity Assay Kit (GPx, CGP1-1KT), and Superoxide Dismutase Determination Kit (SOD, 19160-1KT-F) were all purchased from Merck, Darmstadt, Germany. The IMI used in the experiment is the active compound of a known insecticide (IMI, 100 g·L^−1^) bought from a local market. To ensure transparency and mitigate any potential conflict of interest related to the IMI, the manufacturer’s identity has been kept confidential. This product was deliberately selected for this study to represent an accurate real-life scenario.

#### 3.2.2. Zebrafish Maintenance and Exposure Protocols

**Figure 8:**
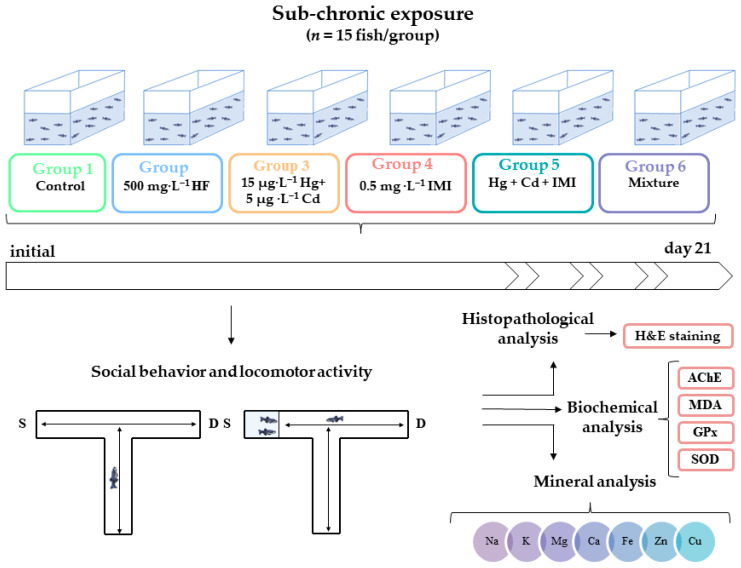
The schematic illustration of the experimental setup (*n* = 15 for each experimental group with two experimental replicates).

Wild-type (AB strain) zebrafish (*Danio rerio*) of both sexes (6–8 months old, 0.38 ± 0.06 g) were obtained from a local supplier and acclimated in the experimental room for two weeks before the experiments. Zebrafish maintenance and experimental procedures were based on previous publications with slight modifications [21,51]. After acclimatization, the animals were randomly separated into the following six groups with 15 animals in each group, as shown in Figure 8: Group 1 (control)—fish were exposed to standard tank water and served as the control group; Group 2 (HF)—fish were exposed to honey enriched with additives at a concentration of 500 mg·L^−1^; Group 3 (Hg + Cd)—fish were exposed to 15 µg·L^−1^ Hg and 5 µg·L^−1^ Cd; Group 4 (IMI)—fish were exposed to 0.5 mg·L^−1^ IMI; Group 5 (Hg + Cd + IMI)—fish were exposed to 15 µg·L^−1^ Hg, 5 µg·L^−1^ Cd, and 0.5 mg·L^−1^ IMI; Group 6 (mixture)—fish were exposed to 15 µg·L^−1^ Hg, 5 µg·L^−1^ Cd, and 0.5 mg·L^−1^ IMI, in combination with 500 mg·L^−1^ HF. According to a previous study [70], a density of approximately 1.59 g of fish per L of water was maintained. Two replicated experiments were carried out for the control and each treatment. During the acclimatization and the 21 days of exposure, the fish were fed twice a day with commercial fish food (TetraMin) at a level of approximately 1% of their body weight per day. Also, the test solutions were renewed every day to maintain the compounds’ concentrations and water quality at stable levels. The concentrations of the heavy metals and pesticide were set based on previous studies [31,37,38,39,42,43,165], while the concentration of HF was selected based on its maximum solubility in water, to achieve a homogeneous and clear aqueous solution of HF.

#### 3.2.3. Behavioral Assessment

Initially and at the end of the 21 days of exposure, zebrafish from control and exposed groups were submitted to behavioral tests to evaluate animal locomotion and social responses (shoal preference). Both tests were performed in a multipurpose T maze (10 cm height × 50 cm length × 50 cm width) filled with water to a height of 5 cm. The fish were allowed to acclimate to the experimental tank for 30 s, after which behavior was video-recorded over a 4 min period.

The video files were recorded with a professional infrared light camera located above the T maze and analyzed with EthoVision^®^ XT 11.5 software (Noldus Information Technology BV, Wageningen, The Netherlands). The software was previously programmed, calibrated, and optimized for the behavioral tests. The total distance traveled (cm) and mean speed (cm·s^−1^) were considered the main parameters of locomotion, whereas the immobility duration (s), characterized in zebrafish by the delay in the swimming activity, represented a parameter of anxiety-like behavior. For the social interaction test, 4 fish from the same experimental group were placed in a compartment in the left arm closed with a transparent slot. During the 4 min session following 30 s of acclimation, the fish could freely swim from the central zone to the other (left zone—social zone; right zone—non-social zone). The analysis of behavior changes was based on a comparison of responses of exposed zebrafish either to responses of unexposed zebrafish (control group) or to responses measured during the pre-exposure period (baseline behavior assessment).

#### 3.2.4. Biochemical Assays

Following the completion of treatment, the zebrafish brains of each group were dissected and homogenized with a KIMBLE^®^ Dounce tissue grinder (Sigma-Aldrich, Saint Louis, MO, USA) by adding 6-fold ice of 0.05 M Tris-HCl with a pH of 8.0, according to the approach of Richetti et al. (2011) [166]. Then, the supernatant was used for the determination of AChE activity, according to a previously reported protocol [22].

For the determination of SOD, GPx activities, and MDA levels, the fish bodies were homogenized with 10 volumes of 0.1 M ice-cold PBS with a pH of 7.4 and then centrifuged (microcentrifuge Biocen 22 R, ORTO ALRESA, Madrid, Spain) for 15 min at 5500 rpm (4 °C). The enzyme activities and MDA levels were measured using test kits according to the manufacturer’s instructions. All measurements were normalized by the determined protein concentration in each sample according to the Bradford method with BSA as a standard.

#### 3.2.5. Mineral Analysis of Zebrafish Whole Body

The concentrations of Cu, Zn, Fe, Ca, Mg, K, and Na were determined following a previously described protocol [22,28,167] and using the same reagents and equipment described in Section 3.1.3. Five fish specimens were selected from each experimental group and subjected to the mineralization process after being washed with ultrapure water.

The validation of the method was performed using a certified reference material (fish muscle, ERM-BB422), which was prepared by applying the same protocol used for experimental samples. The reference material has been certified by the Institute for Reference Materials and Measurements (IRMM) of the European Commission’s Joint Research Centre (JRC). All concentrations are reported as µg·g ^−1^ wet weight.

#### 3.2.6. Histological Analysis and Immunohistochemistry Activity for Brain and Spinal Cord Tissues

After each fish was euthanized using ice-cold water, specimens were fixed in 10% neutral buffered formalin (HT501128, Sigma-Aldrich Chemie GmbH, Taufkirchen, Germany) for 1 h and 48 h in Bouin’s solution (HT10132, Sigma-Aldrich Chemie GmbH, Taufkirchen, Germany) and then dehydrated in different ethanol solutions (Ethanol absolute for analysis, EMSURE^®^, Merck, Darmstadt, Germany), embedded in paraffin, and sectioned at 5 µm thickness using a rotary microtome (Cut 6062, SLEE medical GmbH, Mainz, Germany). Five microscope slides were prepared from each paraffin block and subjected to staining following the standard hematoxylin and eosin (H&E) staining protocol (Sigma-Aldrich, Saint Louis, MO, USA). Immunohistochemistry (IHC) staining was conducted using a series of specific antibodies as markers. Three key regions were thoroughly discussed and analyzed (optic tectum, cerebellum, and spinal cord) with three markers, proliferating cell nuclear antigen (PCNA), S100 beta (S100B), and glial fibrillary acidic protein (GFAP), respectively. These antibodies included GFAP from Synaptic Systems GmbH (Catalog No. 173002, Göttingen, Germany), S100B from Signalway Antibody (Catalog No. C48942, Greenbelt, MA, USA), and PCNA from GeneTex (catalog No. GTX124496, Irvine, CA, USA). For each fish, three slides were subjected to the following procedure: They were initially dewaxed and then microwaved at 95 °C for 10 min in a citrate buffer, pH 6.0, 10×, Antigen Retriever (C9999, Sigma-Aldrich, Saint Louis, MO, USA). Afterward, the slides were allowed to cool for 20 min and subsequently rinsed twice in PBS (P4417, Sigma-Aldrich, Saint Louis, MO, USA) for 5 min each. Next, they were incubated overnight at 4 °C in a humid chamber with primary antibodies. The primary antibodies were diluted to a ratio of 1:1000 for GFAP, 1:100 for S100B, and 1:250 for PCNA. On the following day, the slides were washed three times with PBS for 5 min each and then incubated with secondary antibodies (Goat Anti-Rabbit HRP, ab205718, Abcam, Cambridge, UK) diluted to the same ratio as primary antibodies. Immunohistochemistry sections were developed using a 3,30-diaminobenzidine Substrate Kit (DAB, ab64238, Abcam, Cambridge, UK) and subsequently counterstained with hematoxylin (Merck KGaA, Darmstadt, Germany). Subsequently, they were examined and photographed using an optical microscope (Leica DM750, Leica Microsystems CMS GmbH, Wetzlar, Germany). In addition, the IHC lesion severity scores were calculated as follows: (−) = absent or rarely observed labeling, no lesions; (+) = mild–low lesions (numbers of cells positively labeled up to 10%), (++) = moderate–medium lesions (numbers of cells positively labeled between 10 and 50%), (+++) = severe, major lesions (more than 50% of cells positively labeled) [168].

### 3.3. Statistical Analysis

The data are represented by mean ± standard deviation (SD), and the statistical difference was analyzed either by two-way ANOVA followed by a Tukey’s HSD post hoc test for multiple comparisons between groups or a *t*-test using a statistical analysis software, GraphPad Prism (GraphPad Prism Software, San Diego, CA, USA), version 10.3.1 for Windows. The SigmaPlot software (Systat Software Inc., Erkrath, Germany), version 15 for Windows, was used for the normality test (Shapiro–Wilk), equal variance test (Brown–Forsythe), and the power of the test, with *α* = 0.050. Moreover, a priori sample size calculation was carried out on various studies [44,169,170,171,172] and assuming a one-way between-subjects ANOVA for independent groups to detect a medium to large effect with power exceeding 80% with *α* = 0.05 by using G*Power 3.1.9.7 software (University of Düsseldorf, Germany).

## 4. Conclusions

In our study, we observed that the combined exposure of zebrafish to Cd, Hg, and IMI resulted in a more pronounced impact than any individual treatment alone. Thus, co-exposure to these two heavy metals and IMI over 21 days led to a significant decline in main locomotion parameters and an increase in immobile duration. Exposure to these contaminants adversely affected the social behavior of the fish, with the most substantial reduction in time spent near social stimuli recorded in the group subjected to the ternary mixture. The presence of heavy metals and a pesticide in the habitat of zebrafish resulted in a noticeable decrease in AChE activity within their brains. This decline was accompanied by increased levels of MDA and heightened activity of antioxidant enzymes, such as SOD and GPx, in the fish’s tissues. In addition, the group exposed to the mixture of Cd, Hg, and IMI exhibited excess Ca levels, while elevated Mg levels were noted in the Hg + Cd and mixture groups. In contrast, significant deficiencies in Fe and K were observed in the Hg + Cd and mixture groups. Furthermore, the mixture treatment resulted in degenerative changes in the optic tectum and cerebellum, with relatively milder effects observed in the spinal cord. The results showed that HF co-administration can attenuate the contaminant-induced social and locomotor activity impairments, changes in OS parameters and AChE activity, and brain injury. These supplements hold potential utility within the fish industry for alleviating the adverse effects of environmental contaminants infiltrating aquaculture systems through various pathways. Future research endeavors are warranted to elucidate the precise mechanisms underlying both the toxicological effects induced by concurrent exposure to heavy metals and pesticides and the observed protective effects of health supplements. Furthermore, establishing a dose–response relationship for these supplements remains crucial for advancing our understanding.

## Figures and Tables

**Table 1:** Physicochemical parameters, bioactive compounds, and total antioxidant capacity of honey samples.

Parameters	Honey Sample	
	Polyfloral	Formulation
Physicochemical parameters		
Moisture (%)	19.467 ± 0.115 ^b^	22.667 ± 0.115 ^a^
TSS (°Brix)	80.533 ± 0.115 ^a^	77.333 ± 0.115 ^b^
pH	3.93 ± 0.01 ^b^	4.05 ± 0.05 ^a^
Free acidity (meq·kg^−1^)	20.100 ± 0.100 ^b^	40.067 ± 0.115 ^a^
Electrical conductivity (mS·cm^−1^)	0.453 ± 0.002 ^b^	0.538 ± 0.001 ^a^
Metal ion concentrations (mg·g^−1^)		
Bioelements		
K	238.56 ± 22.24 ^b^	534.63 ± 38.11 ^a^
Ca	63 ± 9.45 ^b^	180.3 ± 19.83 ^a^
Mg	35.53 ± 1.88 ^b^	100.89 ± 5.06 ^a^
Na	5.921 ± 0.51 ^b^	38.084 ± 5.71 ^a^
Zn	11.69 ± 1.25 ^a^	9.31 ± 1.01 ^b^
Fe	5.62 ± 0.5 ^b^	6.52 ± 0.53 ^a^
Mn	0.483 ± 0.053 ^b^	1.139 ± 0.159 ^a^
Cu	0.233 ± 0.028 ^b^	0.513 ± 0.035 ^a^
Cr	0.134 ± 0.012 ^a^	0.143 ± 0.013 ^a^
Toxic metals		
Cd	1.9 ± 0.2 (×10^−3^) ^a^	2 ± 0.3 (×10^−3^) ^a^
Pb	9 ± 2 (×10^−3^) ^a^	10 ± 2.5 (×10^−3^) ^a^
Bioactive compounds		
TPC (mg GAE per 100 g sample)	143.415 ± 1.771	371.318 ± 0.740
TFC (mg RE per 100 g sample)	59.751 ± 0.272	269.932 ± 5.203
TAC (mg CGE per 100 g honey)	1.761 ± 0.127	10.086 ± 0.421
Antioxidant capacity (AC)		
DPPH (mg GAE per 100 g honey)	0.584 ± 0.075	4.851 ± 0.058
ABTS (mg TE per 100 g honey)	1214.72 ± 86.036	2315.716 ± 110.638

Note: Data are expressed as mean ± SD, and values sharing the same letter indicate no significant differences at a confidence level of 95% using the *t*-test.

**Table 2:** Identification and quantification of common phenolic acids, flavonoids, and vitamins in honey samples.

Compound	t_R_ (min)	Control Sample (mg per 100 g Sample)	HF (mg per 100 g Sample)
Phenolic acids			
Gallic acid		Tr	Tr
Pyrogallol	5.94	0.05 ± 0.01	1.76 ± 0.33
Caffeic acid		Tr	Tr
Vanillic acid		Tr	Tr
Syringic acid	8.79	0.78 ± 0.0168	0.27 ± 0.02
p-Coumaric acid		Tr	Tr
Rosmarinic acid		Tr	Tr
Ferulic acid	11.00	0.36 ± 0.01	0.58 ± 0.01
Flavonoids			
Rutin		Tr	Tr
Catechin		Tr	Tr
Quercetin	11.40	0.50 ± 0.01	1.21 ± 0.01
Kaempferol		Tr	Tr
Vitamin			
Riboflavin (Vitamin B_2_)	8.19	0.1 ± 0.01	0.13 ± 0.013
Ascorbic acid (Vitamin C)		Tr	Tr

Note: t_R_—retention time; Tr—traces (below limit of detection).

## Data Availability

Data are contained within the article or Supplementary Materials.

## Supplementary Materials

The following supporting information can be downloaded at: https://www.mdpi.com/article/10.3390/ijms252111730/s1.
