# Supplementary material for: Honey Enriched with Additives Alleviates Behavioral, Oxidative Stress, and Brain Alterations Induced by Heavy Metals and Imidacloprid in Zebrafish
Source: Int J Mol Sci. 2024 Oct 31;25(21):11730. doi: 10.3390/ijms252111730 (PMC11546825; doi:10.3390/ijms252111730)
Supplement: Supplementary file 1 [file ijms-25-11730-s001.zip › ijms-3256348-supplementary.pdf]

International Journal of *Molecular Sciences*

MDPI

Article

Honey Enriched with Additives Alleviates Behavioral, Oxidative Stress, and Brain Alterations Induced by Heavy Metals and Imidacloprid in Zebrafish

Emanuela Paduraru [1†], Roxana Jijie [2†], Ira-Adeline Simionov [3,4], Cristina-Maria Gavrilescu [5], Tudor Ilie [6], Diana Iacob [1], Andreea Lupitu [7], Cristian Moisa [7], Claudia Muresan [7], Lucian Copolovici [7], Dana M. Copolovici [7], Gabriela Mihalache [8], Florin Daniel Lipsa [9], Gheorghe Solcan [10], Gabriela-Alexandra Danelet [10], Mircea Nicoara [1,11], Alin Ciobica [11,12,13,14,*] and Carmen Solcan [10]

1 Doctoral School of Geosciences, Faculty of Geography and Geology, Alexandru Ioan Cuza University of Iasi, No. 20 A Carol I Avenue, 700505 Iasi, Romania; emanuelapaduraru19@yahoo.com (E.P.); dianaelenaiacob84@gmail.com (D.I.)

2 Research Center on Advanced Materials and Technologies (RAMTECH), Department of Exact and Natural Sciences, Institute of Interdisciplinary Research, Alexandru Ioan Cuza University of Iasi, No. 11 Carol I Avenue, 700506 Iasi, Romania; roxana.jijie@uaic.ro (R.J.)

3 Department of Food Science, Food Engineering, Biotechnologies and Aquaculture, Dunarea de Jos University of Galati, No. 47 Domnească Street, 800008 Galati, Romania; ira.simionov@gmail.com (I.-A.S.)

4 REXDAN Research Infrastructure, Dunarea de Jos University of Galati, No. 98 George Coșbuc Street, 800385 Galati, Romania

5 Department of Biomedical Sciences, Grigore T. Popa University of Medicine and Pharmacy, No. 16 University Street, 700115 Iasi, Romania; cristina.gavrilescu@umfiasi.ro (C.-M.G.)

6 Synergy Plant Products, No. 12 Milano Street, Prejmer, 507165 Brasov, Romania; tudoriliebv@gmail.com (T.I.)

7 Faculty of Food Engineering, Tourism and Environmental Protection, Institute for Research, Development and Innovation in Technical and Natural Sciences, Aurel Vlaicu University, No. 2 Elena Dragoi Street, 310330 Arad, Romania; pag.andreea@yahoo.com (A.L.); moisa.cristian@yahoo.com (C.M.); claudia.muresan@uav.ro (M.C.); lucian.copolovici@uav.ro (L.C.); dana.copolovici@uav.ro (D.M.C.)

8 Integrated Center of Environmental Science Studies in the North-Eastern Development Region (CERNESIM), Department of Exact and Natural Sciences, Institute of Interdisciplinary Research, Alexandru Ioan Cuza University of Iasi, No. 11 Carol I Avenue, 700506 Iasi, Romania; gabriela.mihalache@uaic.ro (G.M.)

9 Department of Food Technologies, Ion Ionescu de la Brad University of Life Sciences, No. 3 Mihail Sadoveanu Alley, 700490 Iasi, Romania; florin.lipsa@iuls.ro (F.D.L.)

10 Faculty of Veterinary Medicine, Ion Ionescu de la Brad University of Life Sciences, No. 8 Mihail Sadoveanu Alley, 700489 Iasi, Romania; gsolcan@uaiasi.ro (G.S.); danelet.alexandra95@yahoo.com (G.-A.D.); csolcan@uaiasi (C.S.)

11 Department of Biology, Faculty of Biology, Alexandru Ioan Cuza University of Iasi, No. 20A Carol I Avenue, 700505 Iasi, Romania; mirmag@uaic.ro (M.N.)

12 Center of Biomedical Research, Romanian Academy, No. 8 Carol I Avenue, 700506 Iasi, Romania

13 Academy of Romanian Scientists, No. 54 Independence Street, Sector 5, 050094 Bucharest, Romania

14 "Ioan Haulica" Institute, Apollonia University, No. 11 Pacurari Street, 700511 Iasi, Romania

* Correspondence: alin.ciobica@uaic.ro (A.C.)

† These authors contributed equally.

Citation: To be added by editorial staff during production.

Academic Editor: Firstname Lastname

Received: date
Revised: date
Accepted: date
Published: date

Copyright: © 2024 by the authors. Submitted for possible open access publication under the terms and conditions of the Creative Commons Attribution (CC BY) license (https://creativecommons.org/licenses/by/4.0/).

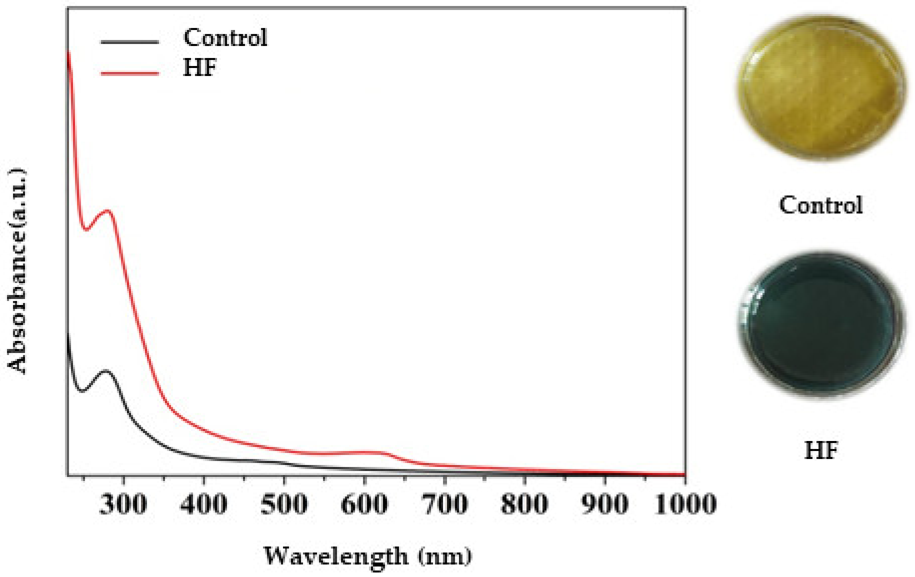

Figure S1. UV-Vis absorption spectra of honey samples and their appearance.

Table S1. The antibiotics used in the experiment.

Bacterial strain	Antibiotic	Concentration
Escherichia coli ATCC 8735	amoxicillin/clavulanic acid (AMC)<br>ceftazidime/clavulanic acid (CXN)	30 μg (20/10)<br>40 μg (30/10)
Salmonella enteritidis ATCC 13076	streptomycin (HLS)<br>cefalexin (CXN)	300 μg<br>30 μg
Pseudomonas aeruginosa ATCC 27853	gentamycin (G)<br>cefalexin (CXN)	40mg·mL^{-1}<br>30 μg
Staphylococcus aureus ATCC 25923<br>*Enterococcus faecalis* ATCC 19433	amoxicillin/clavulanic acid (AMC)<br>clindamycin	30 μg (20/10)<br>2 μg
Listeria monocytogenes ATCC 13932	erythromycin (ERY)<br>streptomycin (HLS)	15 μg<br>300 μg

(A)

(B)

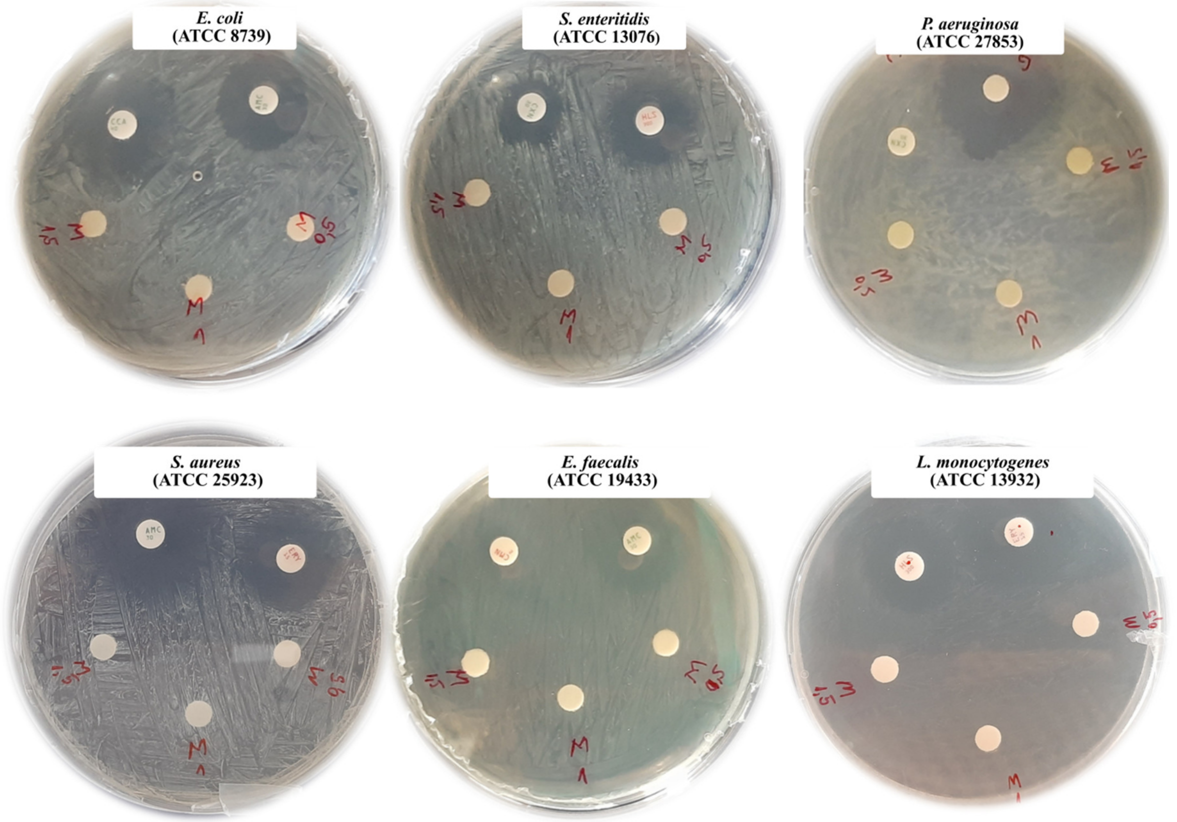

Figure S2. The images obtained by disk diffusion method of the HF and antibiotics against several Gram-negative (A) and Gram-positive bacteria (B).

Table S2. Lesion score and IHC positivity in optic tectum for the control and exposed groups.

Lesions and IHC score	Control	HF	Hg+Cd	IMI	Hg+Cd+IMI	Mixture
Severe spongiosis in the neural layer	-	-	+	+++	+++	++
Edema	-	-	++	+++	+++	++
Vacuolization of the neuropil with multiple areas of extravasated erythrocytes	-	-	++	+++	++	+
Degenerative changes in neurons	-	-	++	+++	+++	++
Focal aggregation of microglial cells	-	-	++	+++	+++	++
Lesion score	-	-	++	+++	+++	++
GFAP score	-	+	+	++	++	+++
S100B score	+	+	++	++	++	+++
PCNA score	++	++	+++	+	++	++

Note: (-) = no lesions; (+) = up to 10%; (++) = between 10 and 50%; (+++) = > 50%.

Table S3. Lesion score and IHC positivity in cerebellum for the control and exposed groups.

Lesions and IHC score	Control	HF	Hg+Cd	IMI	Hg+Cd +IMI	Mixture
Degeneration of neurons	-	-	++	+++	+++	++
Spongiosis in the neuronal layer	-	-	+	+++	+++	++
Vacuolation of gray and white matter with focal aggregation of glial cells	-	-	++	+++	+++	++
Damage to granular neurons	-	-	++	+++	++	+
Lesion score	-	-	++	+++	++	++
GFAP score	+	++	++	++	++	+++
S100B score	+	+	++	++	++	+++
PCNA score	++	++	+++	+	+	++

Note: (-) = no lesions; (+) = up to 10%; (++) = between 10 and 50%; (+++) = > 50%.

Table S4. Lesion score and IHC positivity in spinal cord for the control and exposed groups.

Lesions and IHC score	Control	HF	Hg+Cd	IMI	Hg+Cd +IMI	Mixture
Degeneration of neurons	-	-	++	+++	++	+++
Spongiosis in the neuronal layer	-	-	++	+++	+	+++
Vacuolation of gray and white matter with focal aggregation of glial cells	-	-	++	+++	++	+++
Damage to granular neurons	-	-	++	+++	++	++
Lesion score	-	-	++	+++	++	++
GFAP score	-	++	+++	++	++	++
S100B score	-	+	+++	++	++	++
PCNA score	++	++	++	+	+++	+

Note: (-) = no lesions; (+) = up to 10%; (++) = between 10 and 50%; (+++) = > 50%.
